# Tropical Cyclones and Pediatric Hospitalizations in the US

**DOI:** 10.1001/jamanetworkopen.2025.44013

**Published:** 2025-11-17

**Authors:** Kate Burrows, Samyuktha Natarajan, Chen Chen

**Affiliations:** 1 Department of Public Health Sciences, University of Chicago, Chicago, Illinois; 2 Scripps Institution of Oceanography, University of California, San Diego, La Jolla

## Abstract

This cohort study analyzes the association between short-term exposure to tropical cyclones and all-cause pediatric hospitalization in the US across a 14-day period.

## Introduction

The average Atlantic tropical cyclone (TC) season has 24 storms,^1^ and storm intensity and frequency are projected to increase owing to climate change.^2^ Not all TCs make landfall, but those that do are associated with increases in nonaccidental mortality and morbidity, including mental illnesses and respiratory, infectious, and parasitic diseases.^3^ New data^4^ have facilitated the simultaneous investigation of multiple storms, providing more generalizable findings than single-storm studies. However, multistorm research has largely focused on adults,^3^ despite single-storm studies showing poststorm increases in respiratory symptoms and blood lead concentrations among children, pediatric mental health issues,^5^ and violence against children.^5^ The only multistorm study of TCs and asthma exacerbation among children was inconclusive,^6^ highlighting the need for broader analyses. We analyzed the association between short-term TC exposure and all-cause pediatric hospitalization across a 14-day period.

## Methods

This case-control study used commercial health insurance claims data from 151 metropolitan statistical areas (MSAs) in the US from 2007 to 2018. MSAs were considered exposed if more than 50% of counties in the MSA experienced TCs with gale-force winds or greater (≥34 knots), resulting in 346 MSA storm-days from 2007 to 2018. Of the 35 TCs in the Atlantic Basin during this period, 34 affected MSAs in our dataset. We obtained daily MSA-level counts of all-cause pediatric hospitalizations, stratified by age (0-5 years, 6-12 years, 13-18 years), from the Merative MarketScan Commercial Claims and Encounters Databases. The University of Chicago Institutional Review Board waived the need for ethics review and informed consent owing to the use of deidentified data. We followed the STROBE reporting guideline.

We conducted a case-crossover analysis, comparing storm status between case days and matched control days within the same month, year, day of the week, and MSA. To improve statistical efficiency, we combined matched strata with the same case day into 1 stratum, assigned the total count of hospitalizations during the case day as the weight for the stratum, and conducted a weighted conditional logistic regression. The regression included strata weights of case day hospitalization counts and a 14-day distributed lag structure (lag 0-14). We also conducted stratified analyses across age groups and a Cochran *Q* heterogeneity test. Statistical significance was set at 2-sided *P* < .05. Analyses were performed from September 1, 2024, to September 30, 2025, using R, version 4.4.1 (R Project for Statistical Computing).

## Results

**Figure 1.**
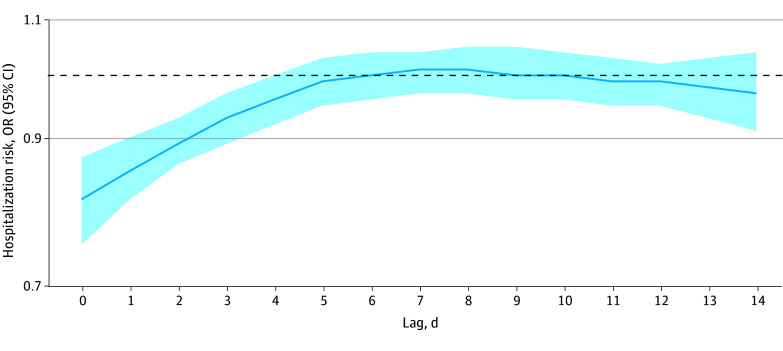
Risk of Pediatric Hospitalizations Associated With Tropical Cyclone Exposure OR indicates odds ratio.

We analyzed data from 991 673 pediatric hospitalizations (51.0% boys, 49.0% girls; 64.0% aged ≤5 years, 11.3% aged 6-12 years, 24.7% aged 13-18 years). Hospitalization risk significantly decreased on storm days (odds ratio [OR], 0.81 [95% CI, 0.75-0.87]), and remained decreased for 3 days (lag 1: OR, 0.85, [95% CI, 0.81-0.90]; lag 2: OR, 0.89, [95% CI: 0.86-0.93]; lag 3: OR, 0.93, [95% CI, 0.89-0.97[) (Figure 1). This decrease was followed by a nonsignificant increase, peaking at 7 to 8 days after a storm. Overall, we observed a cumulative decrease in hospitalizations at 14 days after a storm (OR, 0.75 [95% CI, 0.68-0.83]).

**Figure 2.**
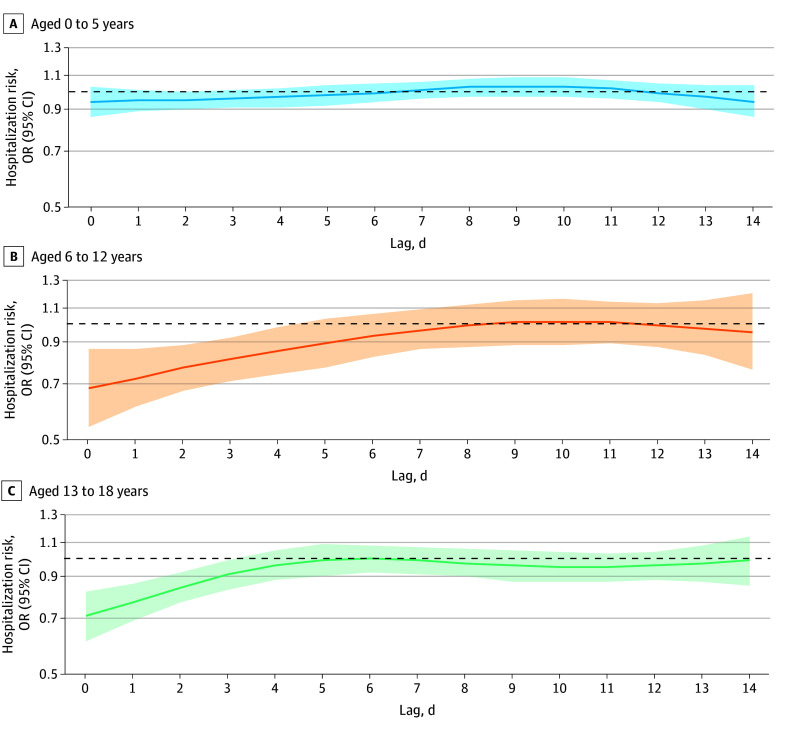
Risk of Pediatric Hospitalizations Associated With Tropical Cyclone Exposure, Stratified by Age Group OR indicates odds ratio.

Heterogeneity across age groups was not significant (*Q* = 5.79, *P* = .06), with greater cumulative reductions observed in older age groups (0-5 years: OR, 0.84 [95% CI, 0.73-0.96]; 6-12 years: OR, 0.60 [95% CI, 0.42-0.87]; 13-18 years: OR, 0.64 [95% CI, 0.51-0.81]). Timing of decreased hospitalizations also varied by age (Figure 2).

## Discussion

Similar to findings for adults, we observed a decrease in pediatric hospitalizations on storm days, likely reflecting behavioral changes due to inclement weather.^3^ These disruptions varied by age group, with marginal heterogeneity and different temporal patterns across groups. Limitations include low pediatric hospitalization rates for some MSA days, limiting our capacity to conduct cause-specific analyses, and the use of private insurance data, which may exclude the most vulnerable children. Future research should assess broad-cause (ie, acute vs chronic disease) and cause-specific hospitalizations to understand underlying mechanisms driving poststorm morbidity. This work is essential to develop targeted interventions and protect children’s health during hurricanes, particularly in the context of climate change.

## Notes

Supplement.
Data Sharing Statement
