# Supplementary material for: Tropical Cyclones and Pediatric Hospitalizations in the US
Source: JAMA Netw Open. 2025 Nov 17;8(11):e2544013. doi: 10.1001/jamanetworkopen.2025.44013 (PMC12625680; doi:10.1001/jamanetworkopen.2025.44013)
Supplement: Supplement. — Data Sharing Statement [file jamanetwopen-e2544013-s001.pdf]

Data Sharing Statement

Burrows. Tropical Cyclones and Pediatric Hospitalizations in the US. *JAMA Netw Open*. Published November 17, 2025. doi:10.1001/jamanetworkopen.2025.44013

Data
Data available: No

Additional Information
Explanation for why data not available: Data was obtained from the Marketscan Commercial Claims and Encounters (CCAE) Database, a national collection of paid inpatient, outpatient, and pharmaceutical claims generated by between 17 and 53 million people per year. The data are collected from over 350 insurance carriers, Blue Cross Blue Shield plans, and third-party administrators. Data was obtained under an agreement between the University of Chicago and Merative and the authors do not have permission to share.
